# Comparison of early and long term outcomes of open Lichtenstein repair and totally extraperitoneal herniorrhaphy for primary inguinal hernias

**DOI:** 10.3906/sag-1803-94

**Published:** 2019-02-11

**Authors:** Barış SEVİNÇ, Nurullah DAMBURACI, Murat GÜNER, Ömer KARAHAN

**Affiliations:** 1 Department of General Surgery, Faculty of Medicine, Uşak University, Uşak Turkey

**Keywords:** Inguinal hernia, Lichtenstein, Laparoscopy, Mesh repair

## Abstract

**Background/aim:**

Inguinal hernia repair is one of the most common surgical procedures worldwide. There is still controversy over which method has the best postoperative results. The aim of this study was to compare early and late postoperative results of laparoscopic totally extraperitoneal herniorrhaphy (TEP) and open Lichtenstein herniorrhaphy (OLR).

**Materials and methods:**

The study was conducted in a randomized prospective manner and it was concluded with 302 patients (147 cases in TEP group and 155 cases in OLR group). All procedures were performed by two experienced surgeons in both open and laparoscopic inguinal hernia repair.

**Results:**

The groups were similar in terms of age, sex, and types of inguinal hernia according to Nyhuss classification. The mean operation time was shorter in TEP group with 49.2 ± 15.5 min vs 54.3 ± 14.6 min in OLR group (P = 0.004). The mean length of hospital stay was significantly shorter in TEP group (P = 0.001). The mean postoperative visual analogue scale score was significantly lower in TEP group. With a mean follow-up of 40.95 months, the recurrence rates were similar in both groups with a rate of 4.3%. In terms of chronic pain, TEP group has better results than OLR with 3.4% vs 25.2%, respectively (P = 0.001).

**Conclusion:**

In experienced hands, TEP procedure has better early and late postoperative results than OLR, whereas recurrence rates are similar.

## 1. Introduction

Inguinal hernia repair is one of the most widely performed surgical procedures worldwide (1). Regardless of the hernia type, the definitive treatment of inguinal hernia is surgery (2). Since the introduction of tension-free herniorrhaphy, the results of the procedure became better and better (3). Open Lichtenstein repair (OLR) is the most widely used technique. In specialized centers, recurrence rate of OLR was reported to be less than 1% (4). The introduction of laparoscopy in inguinal hernia repair began to change the story. At the beginning, there is a learning curve for the laparoscopic totally extraperitoneal (TEP) herniorrhaphy. Therefore, the early studies resulted in controversy (5). Nowadays, with the improvement of both laparoscopic devices and prosthetic materials and the technique; TEP procedure has better results.

After all techniques used in inguinal hernia repair; postoperative pain, time to return to work, recurrences, and chronic pain are the main problems (6). In the literature, laparoscopic procedures seem to be promising for early postoperative results (7). However, to make a decision, there is need for randomized controlled trials with longer follow-up.

The aim of this study was to compare early and long-term results of OLR and TEP procedures in terms of postoperative pain, time to return to work, recurrence, and chronic pain.

## 2. Materials and methods

This randomized prospective study was conducted after local ethical committee approval between January 2012 and January 2015. Patients with inguinal hernia and a desire for surgery were evaluated for the study. Patients with recurrent inguinal hernia, not suitable for general anesthesia or laparoscopy and obstructed or strangulated hernias were excluded from the study. Moreover, to make homogenous groups in terms of incision and pain scores, bilateral inguinal hernias were not included in the study. After informed consent was obtained from the patients, they were randomized into two groups by computer-aided randomization software. 

Primary outcome of the study is long-term pain after surgery. According to previous study data; with a power of 80% and alpha error of 0.05, the groups should consist 153 cases (7). It was planned to include 320 cases (160 cases in each group) for the study. At the follow-up, 18 cases lost follow-up (13 cases in TEP and 5 cases in OLR groups). The study was concluded with 302 cases. 

The first group was accepted as TEP group and the second one as OLR group. All procedures were performed by two surgeons who are both experienced in inguinal hernia repair at both open and laparoscopic procedures with at least 100 TEP procedures. 

Main demographic data, such as age, sex, BMI, ASA score, were recorded preoperatively. At the end of the procedure, operation time and type of the hernia were also recorded. 

### 2.1. Surgical procedures

TEP procedure was conducted under general anesthesia by using the standard technique. A 10 mm torcher was introduced below the umbilicus for camera entrance. After blunt dissection of the preperitoneal area, two 5-mm trocars were introduced at the midline. After dissection of the hernia sac and reduction of the peritoneum, 15 × 12-cm polypropylene mesh was placed and fixed by using titanium fixators to the Cooper’s ligament. After drainage of the CO2 gas, trocars were removed and fascia defects were sutured.

Lichtenstein procedures were performed as the standardized tension-free mesh hernioplasty procedures. Nerves were preserved whenever it was possible. All OLR were performed under spinal anesthesia.

At the postoperative period, the standardized analgesics were administered to the patients. For the first 24 h; paracetamol 500 mg IV was administered every 8 h and metimazole 100 mg IV every 12 h. After the first 24 h, oral 500 mg paracetamol was administered twice a day. Postoperative pain scores were evaluated at 24 h after surgery by using visual analogue scale (VAS). 

Postoperative complications were recorded in the patients’ files. Length of hospital stay was also recorded. 

Patients were called for follow-up visits at 7 and 30 days after the initial procedure. At the early visits, return to daily activity was evaluated and recorded in the patients’ files. Then, they were called at 6 and 12 months, and yearly after that. At follow-up visits, the patients were examined for recurrences, and chronic pain was evaluated. Chronic pain is accepted as the pain started after the procedure and did not disappear for at least three months after the surgery. Pain that affects concentration on daily activities is defined as severe pain. 

Statistical analysis was performed by using IBM SPSS Statistics 20.0 software. For the qualitative variables, chi-square test was used. When comparing the means of quantitative variables Student’s t-test was used. A P-value lower than 0.05 is accepted as statistically significant.

## 3. Results

The study was concluded with 302 participants. The mean age of the cases was 48.2 ± 13.6; male to female ratio was 273/29. The groups were similar in terms of age and sex (P = 0.115 and 0.408, respectively). Types of inguinal hernias according to Nyhus classification were also similar in between the groups (Table 1).

**Table 1:** Demographics and types of inguinal hernias according to groups.

		TEP	OLR	Total	P
Age		46.9 ± 14.1	49.4 ± 13	48.2 ± 13.6	0.115*
Sex		135/12	138/17	273/29	0.408**
Nyhus type	1	75 (51%)	74 (47.7%)	149 (49.3%)	0.820**
	2	48 (32.7%)	57 (36.8%)	105 (34.8%)	
	3a	16 (10.9%)	14 (9%)	30 (9.9%)	
	3b	8 (5.4%)	10 (6.5%)	18 (6%)	
Follow-up		42.44±11.3	39.5±22.4	40.95±17.9	0.160*

*Student’s t-test, **Chi-Square test. TEP: Laparoscopic totally extraperitoneal herniorrhaphy. OLR: Open Lichtenstein repair.

The mean follow-up time of the study was 40.95 ± 17.9 months and the groups were similar (P = 0.160).

The mean operation time was shorter in TEP group with 49.2 ± 15.5 min vs 54.3 ± 14.6 minutes in OLR group (P = 0.004). Postoperative 24-h VAS score was also significantly lower in TEP group with a mean score of 2.24 ± 1.1 vs 2.64 ± 1.3 (P = 0.005) (Table 2). 

**Table 2:** Distribution of length of hospital stay, return to work, operation time, and 24th hour VAS scores according to groups.

	TEP	OLR	Total	P*
LOS	1.05 ± 0.256	1.25 ± 0.530	1.16 ± 0.43	0.001
Return to work	8.67 ± 2.47	9.17 ± 2.4	8.93 ± 2.4	0.08
Operation time	49.2 ± 15.5	54.3 ± 14.6	51.8 ± 15.2	0.004
VAS score	2.24 ± 1.1	2.64 ± 1.3	2.44 ± 1.2	0.005

*Student’s t-test. LOS: Length of hospital stay. VAS: visual analogue scale. TEP: Laparoscopic totally extraperitoneal herniorrhaphy. OLR: open Lichtenstein repair.

The overall mean length of hospital stay was 1.16 ± 0.43 days (Table 2). It was significantly shorter in TEP group with 1.05 ± 0.2 days vs 1.25 ± 0.5 days, respectively (P = 0.001). The overall mean time to return to work was 8.93 ± 2.4 days and this time was similar in both groups (P = 0.08). 

In TEP group, the main complication was pneumoperitoneum. It occurred in 6 cases (4%). In none of those cases, there was a need for conversion to open or trans-abdominal surgery. At the postoperative period, there was no wound complication. In OLR, there were wound complications in 12 cases (7.7%). We observed seroma in 6 (3.8%) cases, hematoma in 4 (2.5%) cases, and wound infection in 2 (1.2%) cases. All of these patients were treated conservatively.

The overall recurrence rate was 4.3% (Table 3) and it was found to be similar in both groups with 3.4% and 5.2%, respectively (P = 0.451). The rate of chronic pain was significantly lower in TEP group (P = 0.001). The rate of chronic pain in TEP group was 3.4% while it was 25.2% in OLR group. Since the high rate of chronic pain was defined by the patients, we ask the patients another two questions “Does your pain affect your daily activities?” and “Do you really feel pain or just a discomfort because of the hard material at the operation side?”. The rate of pain affecting daily activities was found to be 0.7% in TEP group and 1.3% in OLR group (P > 0.05).

**Table 3:** Distribution of recurrence, time of recurrence, and chronic pain according to groups.

	TEP	OLR	Total	P
Recurrence	5 (3.4%)	8 (5.2%)	13 (4.3%)	0.451*
Chronic pain	5 (3.4%)	39 (25.2%)	44 (14.6%)	0.001*
Time of recurrence	15 ± 8.8	18.3 ± 5.1	17 ± 6.6	0.4**

*Chi-Square test, **Student’s t-test. TEP: Laparoscopic totally extraperitoneal herniorrhaphy. OLR: open Lichtenstein repair.

## 4. Discussion

The present study compared the early and late results of TEP and OLR for inguinal hernias with an acceptable population number and follow-up time. The findings of our study showed that TEP procedure has better results in terms of postoperative acute and chronic pain with similar recurrence rates.

The present study showed shorter operation time in TEP group. Studies comparing the operation time between TEP and Lichtenstein generally reveal lower operation time in OLR (8,9). Dhankhar et al. reported 11 min shorter operation time in OLR compared to TEP group (10). Our results seem to be controversial with those studies; however, with the experience in laparoscopic surgery and laparoscopic groin anatomy, the operation time can be reduced significantly. In our study, all operations were performed by two experienced laparoscopic surgeons; moreover, there was no conversion to open surgery. 

In the present study, we found lower postoperative pain scores in TEP group (2.24 ± 1.1 vs 2.64 ± 1.3). This result shows similarity with the literature (11). Lower pain scores should be associated with smaller incision in TEP group. However, according to Westin et al., local anesthesia use during OLR reduced postoperative pain significantly. They reported no significant difference in postoperative pain after TEP and OLR. As local anesthetics use is not a routine application in OLR, in our study, we did not use local anesthetics in OLR. 

Postoperative hospital stay was found longer in OLR (1.05 ± 0.256 vs 1.25 ± 0.530). The longer hospital stay can be associated with wound complications seen in OLR group. There was no wound complication in TEP group and most of the cases were discharged on the first postoperative day. With respect to our findings; smaller incision means fewer complications. Our wound complication rate is in accordance with those in the literature (10).

In the present study, time to return to work was found similar in both groups (8.67 ± 2.47 vs 9.17 ± 2.4 days). In a metaanalysis, time to return to work was found to be significantly shorter in TEP group (12). The reason for the similar results of the groups in our study is that we limited physical exercise in both groups for a week. 

Chronic pain was defined as pain lasting more than 3 months after surgery. Rate of chronic pain was significantly higher in OLR group (3.4% vs. 25.2%). In terms of chronic pain, there are controversial reports in the literature. According to Dhankar et al. postoperative pain scores were similar in both groups (10). However, they also found higher analgesic use in OLR group. In a metaanalysis, chronic pain was reported to be significantly higher in OLR (12). Our results are in accordance with the findings of the metaanalysis. The main accepted reason for higher chronic pain rate in OLR is the association of the mesh with the inguinal nerves. Although nerves are usually preserved at the OLR, postoperative inflammation of the area may cause chronic pain. Moreover, placement of the mesh at preperitoneal area can be seen as a protective factor against chronic pain. Therefore, we tried to differentiate pain from discomfort of the prosthetic material. The rate of pain affecting daily activities was found to be 0.7% in TEP group and 1.3% in OLR group (P > 0.05). The groups are found to be similar in terms of severe pain. The majority of the cases defining chronic pain claimed only discomfort of prosthetic material.

With a follow-up of 40.9 months, we observed recurrence in 3.4% of cases in TEP group and 5.2% of cases in OLR group. There was no significant difference in terms of recurrence rates. According to Bobo et al. the recurrence rates of the both operations are similar if the follow-up is less than 3 years (12). They suggest that with longer follow-up time recurrence of the TEP group is higher. Similarly, Koning et al. reported higher recurrence in TEP group (6). Langeveld et al. reported similar recurrence rates with a follow-up of 49 months (13). The mean follow-up time of our study is enough to make a decision. Moreover, the mean time of recurrences was 17 months and we did not observe any recurrences after the 28th month. With more experience, recurrence rates of TEP procedure can be reduced. 

Main limitation of our study is that we did not include bilateral or recurrent cases. Inclusion of those cases would make heterogeneity between the groups and could affect the results. We wanted to evaluate homogeneous groups of hernias. The difference in type of anesthesia used in TEP and OLR can be another limitation. However, all around the world the gold-standard anesthesia type for OLR is accepted as spinal anesthesia. Therefore, we did not change the type of anesthesia in OLR. Moreover, the study was not blinded, because the incisions of both groups are different and it is difficult to make any kind of blinding in that case. 

In our study, all cases were operated by only two surgeons with similar experience on both OLR and TEP with at least 100 TEP procedures. Therefore, there is no surgeon-dependent difference between the groups. 

In conclusion, our study showed that in experienced hands, TEP procedure has better results in terms of postoperative pain, length of hospital stay, and chronic pain with similar recurrence rates as OLR.
